# Punding Behavior as a Red Flag for Dementia in a Patient With Depression: Case Report

**DOI:** 10.3389/fpsyt.2021.637861

**Published:** 2021-04-12

**Authors:** Arnaud Pouchon, Clément Dondé, Mircea Polosan

**Affiliations:** Univ. Grenoble Alpes, Inserm U1216 Grenoble Institute of Neurosciences, Psychiatry Department CHU Grenoble Alpes, Grenoble, France

**Keywords:** punding, abnormal movement, dementia, frontotemporal dementia, depression, mood disorder, brain perfusion SPECT

## Abstract

Punding is defined as a stereotypic, complex, repetitive, and non-goal-oriented activity. This behavior has been observed in Parkinson's disease and chronic amphetamine users. However, in general, punding behavior is largely under-diagnosed. Here, we describe a rare case of a 53-year-old woman showing punding behavior during major depressive disorder with atypical clinical features suggestive of a frontal syndrome. Neuropsychological evaluations mainly reported deficits in executive functioning. Brain MRI and lumbar puncture were normal. Brain perfusion SPECT showed hypoperfusion predominating in the right frontal and parietooccipital lobes, and a slight hypoperfusion in subthalamic nucleus including the posterior area of right striatum. We diagnosed this case as a frontotemporal dementia. Punding behavior could be a red flag for dementia in patients with major depressive disorder.

## Introduction

The medical term ≪punding≫ was introduced by Friedman in 1994. He described a 65-years old man with a Parkinson's disease (PD) treated by levodopa and presenting with odd, unusual behaviors [e.g., he spent inordinate amounts of time repetitively adding up tables of numbers with no obvious reason; (1)]. Punding is defined as a stereotypic, complex, repetitive and non-goal-oriented activity (2, 3). This symptom is associated with an intense fascination to common objects, which are constantly manipulated, examined, collected, sorted, and stored by the patient that suffers from punding. These repetitive behaviors appear similar to, and often mimic, previous behavioral habits [i.e., “idiosyncratic”; (4)]. Critically, punding behaviors occur at the expense of functional behaviors which can lead to negative psychosocial impact in daily life (3). Punding behavior has been mostly reported in chronic amphetamine (5) and cocaine users (6), and in PD patients treated by dopamine agonists (2). In parallel, punding was anecdotally described in patients after stroke disease and with bipolar disorder (7). Here, we present a rare case of a patient with major depression associated with punding behavior predictive of dementia.

## Case Report

A 53-year-old woman was referred to our mood disorder clinic for treatment-resistant depression. Her past medical history includes mitral valve prolapse and hypercholesterolemia, treated by pravastatin sodium 20 mg/d. She had a first major depressive episode at 48 y.o., which was successfully treated with paroxetine 20 mg/d. Her current symptoms started 1 years before with suicidal ideation, abulia, anhedonia, emotional blunting, sleep disorder, followed by anorexia with rapid weight loss of 26 pounds, which led to her hospitalization 8 months later. Her current clinical assessment revealed a Montgomery-Asberg Depression Scale score of 18/60. Her cerebral MRI and blood sample were normal excepted hypercholesterolemia. In addition, atypical clinical features suggestive of a frontal syndrome were observed, including psychomotor agitation, ideomotor apraxia, disinhibition, verbal aggression, verbal repetitiveness, unusual urge to smoke and eat, and ideomotor perseverations. Specifically, she devoted a significant amount of time sorting, storing and counting her cigarettes. Obsessive Compulsive Disorder was ruled out since no obsessions were noticed and repetitive behaviors aimed not at relieving anxiety. These atypical behaviors were thus recognized as punding in this patient.

**Figure 1:**
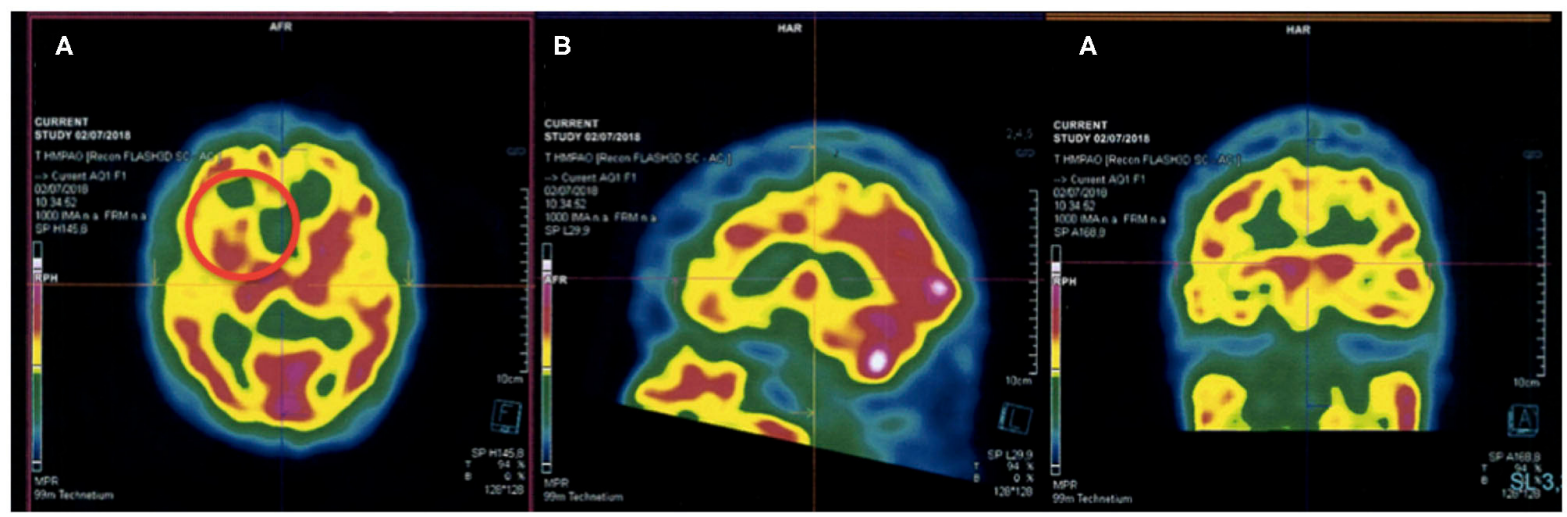
Brain perfusion SPECT showing **(A)** hypometabolism of the right dorsal striatum (red circle), **(B)** hypometabolism of the frontal lobes, and **(C)** hypometabolism of parieto-occipital lobes.

Treatment of the current depressive episode started with a course of venlafaxine 150-mg/d, which was replaced by sertraline 100 mg/d due to poor blood pressure tolerance. Notably, extrapyramidal symptoms quickly emerged after introduction of sedative antipsychotic treatment (loxapine 50 mg/d). Neuropsychological evaluations reported deficits in memory and executive functioning (Table 1). The patient progressively achieved remission of depressive symptoms after 6-month treatment. However, frontal symptoms with a predominance of punding behavior persisted. These features gave rise to suspicion of dementia so further investigations were carried out. The lumbar puncture ruled out an Alzheimer's disease. Repeated neuropsychological examination showed memory improvements associated with remission of depressive symptoms, while altered executive functioning was still present at evaluation (Table 1). Brain perfusion single photon emission computed tomographic (SPECT) showed hypoperfusion predominating in the right frontal and parietooccipital lobes, as well as a slight hypoperfusion in subthalamic nucleus including the posterior area of right striatum (Figure 1). Accordingly, we diagnosed with neurologists this case as a frontotemporal dementia. Afterwards, progressive worsening of autonomy strengthens this hypothesis.

**Table 1:** Clinical evaluation and main results of neuropsychological examination at baseline and 7 months later.

	**Symptom/test**	**Baseline**	**7-month**
Clinical	MADRS	18/60	10/60
	MMSE	20/30	24/30
	Attention deficits	+	+
	Perseverations	+	+
	Psychomotor retardation	+	+
	Automatic behavior dysfunction	+	+
	FAB	13/18	07/18
	Five-word test	10/10	07/10
	Clock drawing test	30/30	26/30
Executive function	STM (Memory span)	4 (reverse: 2)	4 (reverse: 2)
	Semantic fluency test	7 (−2.7σ)	9 (−3.1σ)
	Phonemic fluency test	9 (−1.6σ)	11 (−2.12σ)
	TMT A	2.51 min (+0σ)	1.46 min (−4.5σ)
	TMT B	Fail	4 min (−4.31σ)
	Inhibition (STROOP)	NA	13–20 (−1.6/−2σ)
Memory function	Orientation - Time - Space	4/5 4/5	4/5 5/5
	Recognition	16/16	16/16
	Free recall	14/48 (29%)	23/48 (48%)
	Cued recall	34/48 (71%)	39/48 (81%)
	Delayed recall	13/16 (75%)	15/16 (87.5%)

*NA, not available; +, present; MMSE, mini-mental state examination; FAB, frontal assessment battery; MADRS, Montgomery-Asberg Depression Rating Scale; STM, short term memory; TMT, trail making test*.

Regarding our case, we can expect a progressive pejorative evolution over several years with a worsening of the symptomatology and a progressive loss of autonomy. To date, no specific treatment is available for this disease. Psychotropic medication should be adjusted according to the progression of symptoms. In parallel, non-pharmaceutical approaches should be offered to the patient, including physiotherapy, psychotherapy and family support besides, neurology monitoring should be continued in a Memory clinic. In the event of too great a loss of autonomy, the patient can be placed in an institution.

## Discussion

This case report describes punding behavior in a patient presenting initially with major depressive disorder associated with atypical frontal features, which eventually led to the diagnosis of frontotemporal dementia.

To the best of our knowledge, there is only one report of punding behavior in early onset dementia (8). Authors described the case of a patient with abnormal behaviors including punding with no other motor signs, associated with memory impairment and abulia. The main clinical hypothesis was a major depressive disorder driving cognitive impairment at foreground, before shifting toward Alzheimer's disease. According to the authors, etiological diagnosis of punding is complex and several dementia hypotheses could be envisaged when punding, including Alzheimer's disease, frontotemporal dementia, vascular, or mixed dementia.

Mechanistically, punding has been linked to hyperdopaminergic activity after observation of this behavior in PD patients treated with agonist dopaminergic agents. It is thought that punding is secondary to an excessive stimulation of dopamine receptors in the mesolimbic pathway (4, 9), which, as mediated by glutamatergic adaptation of cortico-striatal circuit, can hamper the striatal structure and function (10). Moreover, it has been hypothesized that punding may be related to plastic changes in the dorsal and ventral striatal structures (7). This model is supported by cortico-striatal disconnections inducing reward-seeking disruption and, in turn, repetitive and stereotypic behavior in both healthy and clinical populations (11, 12). In parallel, it was shown that PD patients with punding present with profound deficits in frontal executive functions and severe cortical thinning in the dorsolateral prefrontal and orbitofrontal area, suggesting prefrontal alterations as a critical component in the development of punding (13). From a clinical perspective, punding has been included in the broader “dopamine dysregulation syndrome” which is frequently observed in PD patients, and which includes pathological gambling, compulsive buying, and hypersexuality (9, 14). An alternative view suggests that punding is a repetitive behavior that was primarily functional and goal-directed and became a habit with a lack of voluntary control (7). Hence, we can speculate that in our patient, who previously consumed cigarettes and worked as tobacconist, cigarette-related behavior was initially seeking for nicotine benefits (e.g., relief, social norming) and became “automatic” or “habitual.” According to the brain perfusion SPECT, a dorsal hypoperfusion of the striatum was observed in our case. This subcortical area is involved in selection and initiation of movement (10), and it is a possibility that decreased connectivity between the striatum and cortical regions involved in motor control led to automatic movements without voluntary control, i.e., punding, in our case (4, 7, 10). An important limitation is that we reported a single case, which corresponds to a low level of evidence, and the observed findings could simply be due to coincidence. Further studies involving larger clinical samples are warranted to better study the predictive value of punding in depression.

In conclusion, it appears relevant to investigate dementia in patients with depression associated with atypical behavioral symptoms such as punding.

## Data Availability Statement

The original contributions generated for the study are included in the article/supplementary material, further inquiries can be directed to the corresponding author/s.

## Ethics Statement

Written informed consent was obtained from the individual(s) for the publication of any potentially identifiable images or data included in this article.

## Author Contributions

AP: formal analysis, investigation, roles/writing—original draft, writing—review, and editing. CD and MP: conceptualization, methodology, project administration, supervision, validation, visualization, writing—review, and editing. All authors contributed to the article and approved the submitted version.

## Conflict of Interest

The authors declare that the research was conducted in the absence of any commercial or financial relationships that could be construed as a potential conflict of interest.
